# Functionalized Hyaluronic Acid for “*In Situ*” Matrix Metalloproteinase Inhibition: A Bioactive Material to Treat the Dry Eye Sydrome

**DOI:** 10.1021/acsmacrolett.2c00455

**Published:** 2022-09-14

**Authors:** Susi Burgalassi, Marco Fragai, Oscar Francesconi, Linda Cerofolini, Daniela Monti, Gemma Leone, Stefania Lamponi, Giuseppe Greco, Agnese Magnani, Cristina Nativi

**Affiliations:** † Department of Pharmacy, University of Pisa, via Bonanno 6, 56126 Pisa, Italy; ‡ Department of Chemistry (DICUS), University of Florence, Sesto Fiorentino 50019, Italy; § CeRM, via Sacconi 6, Sesto Fiorentino 50019, Italy; ∥ CIRMMP, University of Florence, via Sacconi, 6 Sesto Fiorentino 50019, Italy; ⊥ Department of Biotechnology, Chemistry and Pharmacy, via A. Moro, 2 53100 Siena, Italy; # Rugani Hospital, SR222 Chiantigiana, 53035 Colombaio (Siena), Italy; @ INSTM, via G. Giusti, 9, 50121 Firenze, Italy

## Abstract

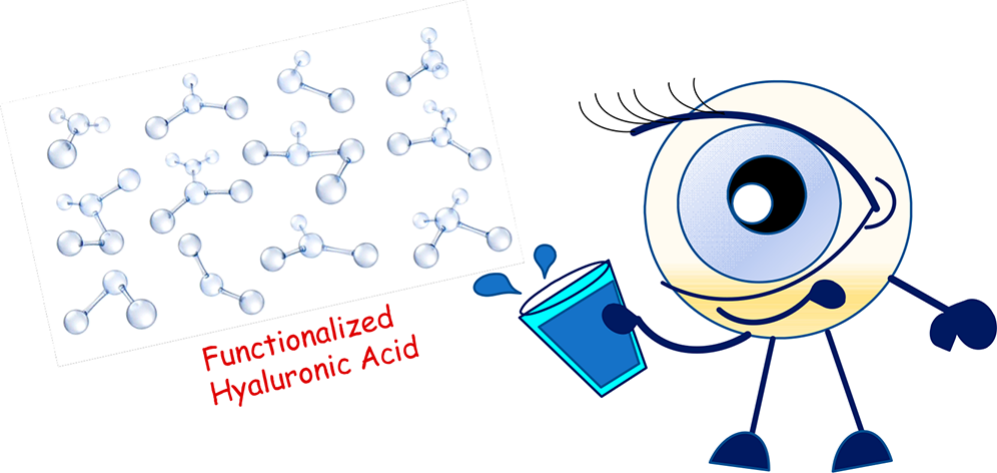

Hyaluronic acid (HA) is a naturally occurring polysaccharide with many molecular functions, including maintaining the structure and physiology of the tissues, tissue remodeling, and inflammation. HA is found naturally in physiological tear fluid, possesses excellent mucus-layer-adhesive properties, and is successfully employed in the treatment of dry eye syndrome (DES). However, HA has as major drawback: its rapid *in vivo* degradation by hyaluronidase. We report on a unique material, namely, HA-**3**, obtained by the functionalization of HA with the metalloproteinase inhibitor **3** (MMPI). This material is characterized by an increased resistance to hyaluronidase degradation, associated with MMP inhibition properties. The ability of HA-**3** to prevent dehydration of human corneal epithelial cells *in vitro* and *in vivo* may accelerate the development of more efficient DES treatment and broaden the application of HA in human diseases.

The highly diversified biochemical and biological roles of metalloproteinases (MMPs) have been known and investigated since the end of the 20th century. Their major action is extracellular matrix (ECM) remodeling; thus, it is not surprising that MMPs are widespread in most connective tissues. However, MMPs have also been localized in many cell types (i.e., endothelial, vascular, and muscular), suggesting this family of proteins is also involved in cell signaling and molecular pathways.^1^

MMPs are a family of Zn^2+^-containing endopeptidases. In human tissues the 23 different MMPs currently identified are structurally highly conserved. A major difference is in the S1′ hydrophobic pocket, located near the enzyme catalytic domain, which presents different depths and dimensions and affects MMP–substrate specificity.^2^

Under physiological conditions, MMPs are essential for the maintenance of healthy states. Conversely, the overproduction of active MMPs, due to an imbalance of natural MMP inhibitors (i.e., tissue inhibitors of MMPs and TIMPs), correlates to disease initiation and progression.^3^

In past years, the development of MMP inhibitors (MMPIs) has represented a promising therapeutic approach to counterbalance the abnormal activation of MMPs, and a plethora of efficient synthetic compounds have been reported.^4−8^

However, the low selectivity affecting all the inhibitors proposed, along with their poor physiological solubility and bioavailability, caused the failure of the clinical trials conducted.^9,10^ Thus, MMP inhibition was classified as an elusive task, and synthetic MMPIs lost therapeutic interest.^11^

Some years ago, we developed a new family of MMPIs,^12^ structurally related to the nanomolar inhibitor NNGH^13^ but, unprecedentedly, soluble in water (Figure 1). For example, Figure 1 shows inhibitors **1** featuring a hydrogen (**1a**) or a polar group (**1b**, **PES_103**) replacing the apolar *sec*-butyl residue displayed by **NNGH** and conferring water solubility. As we showed, polar groups do not affect the affinity of the inhibitors (in the nanomolar range) vs a panel of MMPs (Figure 1).^14,15^ Although innovative, water-soluble MMPIs enable us to overcome the problem of bioavailability, but they do not address the lack of selectivity.

**Figure 1:**
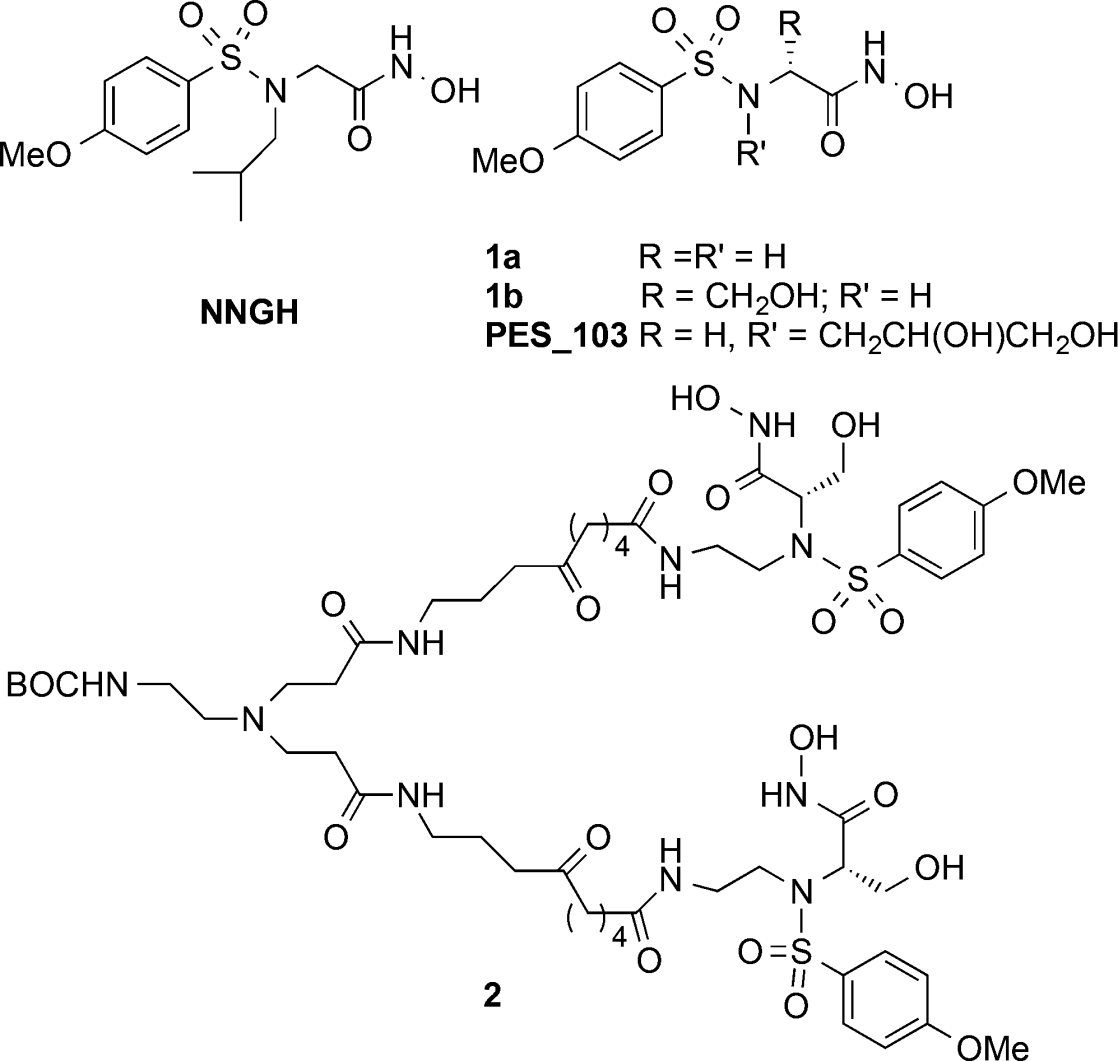
Structure of **NNGH**, of water-soluble inhibitors **1a**,**b** of **PES_103**, and of PAMAM-based inhibitor **2**.

In recent years, exosite targeting inhibitors, neutralizing antibodies, or molecules able to inhibit MMP interactions with cell surface binding counterparts have been proposed as workarounds to selectively modulate MMP activity.^11^ Topical application of MMPIs is another strategy successfully used to overcome inhibitors’ lack of selectivity.^16^ In this context, we have proved the efficacy of water-soluble inhibitors **PES_103** and **2** in “*in situ*” treatment of dry eye syndrome (DES), an orphan pathology characterized by an increase of MMP-9 expression.^17,18^ In particular, we reported the effectiveness and therapeutic potential of the PAMAM-based divalent MMP inhibitor **2** (Figure 1) when locally administered in an experimental model of dry eye.^18^

Undoubtedly, the inhibition of locally overexpressed, detrimental MMPs is an effective strategy to overcome problems associated with their indiscriminate inhibition. Nonetheless, under these circumstances, the possible tissue absorption of the locally administered inhibitor is a major concern. Moving a step forward, in this manuscript we propose a nontoxic new hyaluronan as a bioactive material to treat DES “*in situ*”.

Hyaluronic acid (HA), a naturally occurring polysaccharide consisting of the repetition of a disaccharide composed of d-glucuronic acid (GLCA) and *N*-acetyl d-glucosamine (GlcNAc), plays a role in numerous molecular functions that contribute to the structure and physiology of the tissues, modulating cell behavior during morphogenesis, tissue remodeling, and inflammation.^19,20^ HA is found naturally in physiological tear fluid and possesses excellent moisturizing and mucus-layer-adhesive properties. The inherent biocompatibility together with the susceptibility to chemical modifications have made HA particularly attractive for the development of viscoelastic tools with a broad clinical potential, including ophthalmology.^21,22^ HA has been studied extensively for its applications for the treatment of DES. Dry eye is the disease of the tears and ocular surface that results in symptoms of discomfort, visual disturbance, and tear film instability.^23^ Between 5 and 34% of people are affected by dry eye, with symptoms ranging from redness, burning, stinging, foreign body sensation, pruritus, and photophobia.^24^ Currently, the most popular therapy to treat DES is the use of artificial tears made up of poly(vinyl alcohol), povidone, hydroxypropyl guar, cellulose derivatives, and HA. These components collectively have been shown to increase tear film stability, reduce surface stress, and improve contrast sensitivity and optical surface quality.

HA possesses excellent viscoelastic properties that can lubricate the ocular surface, reducing friction during blinking and ocular movements.^25^ Thus, the water retention and lubricant properties of HA are applied directly to the benefit of dry eye.^26^ The major drawback of HA is its rapid *in vivo* degradation by hyaluronidase. However, cross-linking and functionalization are reported to increase the resistance to HA against enzymatic degradation by hyaluronidase.

In this scenario, the proposed unique material, obtained by the covalent functionalization of hyaluronic acid with the nanomolar inhibitor **3** (Figure 1 and Scheme S1), is characterized by an increased resistance to hyaluronidase degradation, associated with MMP inhibition properties. In addition, since **3** is covalently linked to the polysaccharide, no release of the inhibitor can occur.

Inhibitor **3** was efficiently synthesized^27^ (see SI) and properly armed to be linked to hyaluronic acid (see SI).^28^

Since DES is characterized by an ocular overexpression of MMP-9, the inhibition property of **3** vs MMP-9 (*K*_*i*_ = 16.4 ± 1.7 nM) was assessed by an enzymatic assay (see SI for details). We also evaluated the interaction of inhibitor **3** with the catalytic domain of MMP-12 (selected as model MMP) by NMR (Figure S1, Supporting Information). 2D ^1^H–^15^N HSQC NMR spectra were recorded on a sample of ^15^N isotopically enriched MMP-12 in the absence and presence of an equimolar concentration of **3**. The analysis of the residues experiencing the largest effects proved that the binding of the inhibitor **3** occurs at the active site and involves the amino acids usually affected by the arylsulfonamide scaffold^14,15,29^ (Figure S1). The 2D ^1^H–^15^N HSQC NMR experiments were recorded on a sample of an ^15^N isotopically enriched MMP-12 catalytic domain at the concentration of 0.1 mM, in 10 mM Tris-HCl buffer with 10 mM CaCl_2_, 0.1 mM ZnCl_2_, 0.3 M NaCl, 200 mM acetohydroxamic acid at pH 7.2, and 10% D_2_O. The measurements were performed at 298 K on a Bruker AVANCE III 950 MHz spectrometer, before and after the addition of an equimolar concentration of the inhibitor **3** dissolved in DMSO-*d*_6_.

The functionalization of hyaluronic acid (MW 2000 kDa) was performed as previously reported.^28^ The synthesis is however briefly described in the Supporting Information (Scheme S1).

The chemical composition, the rheological properties, the nontoxicity, as well as the inhibition properties vs MMPs and the increased enzymatic stability *in vitro* of the new material with respect to the native hyaluronan has been proved by physicochemical tests, as previously reported.^28^

The ability of HA-**3** to prevent dehydration of human corneal epithelial cells was first investigated by an *in vitro* test and compared to a commercial product (OPTO yal A – Sooft Italia S.p.A.). As shown in Figure 2, the treatment of HCECs (human corneal epithelial cells) with the commercial tear substitute (OPTO yal A) and the HA-**3** derivative reduces the viability of cells after exposure of the cell monolayer to 30 min of continuous air flow compared to untreated cells (complete medium), but to a significantly lesser extent than with PBS. Furthermore, cell viability after 30 min of contact with OPTO yal A is not statistically different from that observed after contact with HA-**3**. This result is important considering that the commercial product which contains amino acids, in addition to hyaluronan that positively contributes to the lubricating effect of the polysaccharide, shows the same effectiveness as HA-**3**.

**Figure 2:**
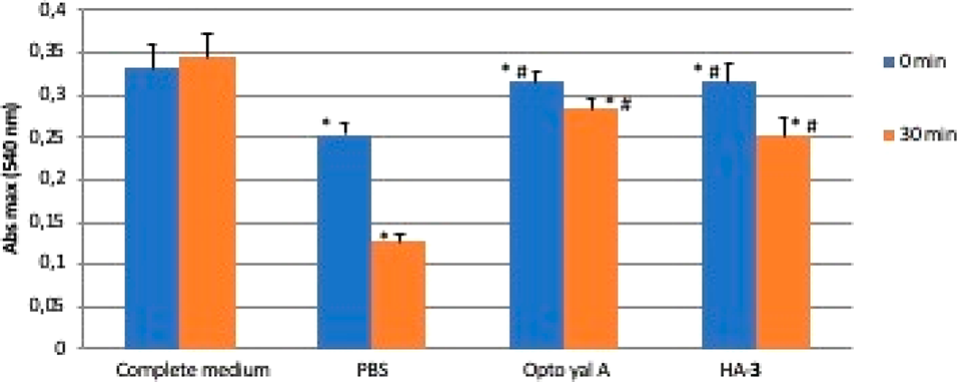
Viability of HCECs after contact with test samples and exposure to continuous airflow for 0 and 30 min. Each sample was tested in triplicate. Complete medium: cells not subject to air flow. PBS: cells in contact with PBS for 20 min and subsequently exposed to the air flow for 0 and 30 min. OPTO yal A: cells in contact with the tear substitute for 20 min and subsequently exposed to the air flow for 0 and 30 min. HA-**3**: cells in contact with the HA-**3** derivative for 20 min and subsequently exposed to the air flow for 0 and 30 min. *Values are statistically different versus complete medium, *p* < 0.05. ^#^Values are statistically different from PBS.

The activity of functionalized HA, HA-**3**, on DES was then tested on rabbits in an experimental model that is accompanied by the increase in MMP-9 expression.^30^ The Schirmer test scores (reported as millimeters of wet strip 3 min after insertion) obtained before (basal values) and after (dry eye) treatment with AS, and relevant to the treatment with the formulation under test, are reported in Figure 3. A decreasing trend of the tear production was observed after beginning AS administration, even if statistically different (*p* < 0.05, unpaired *t* test with Welch’s correction) from the basal value (17.19 mm) only at the fourth and fifth day of treatment. Eyes treated with HA-**3** showed greater scores with respect to control dry eyes at all experimental times, with values of about 20 mm and with statistically significant differences on the third, fourth, and fifth days of treatment (*p* < 0.05, unpaired *t* test with Welch’s correction). Despite that antimuscarinic drug administration causes reduction of tear secretion, functionalized HA is able to maintain the normal hydration degree on the corneal surface. We believe that this ability is due to the well-known property of the HA of water being retained by virtue of spreading as a film over the cornea during the blinking.^31−33^

**Figure 3:**
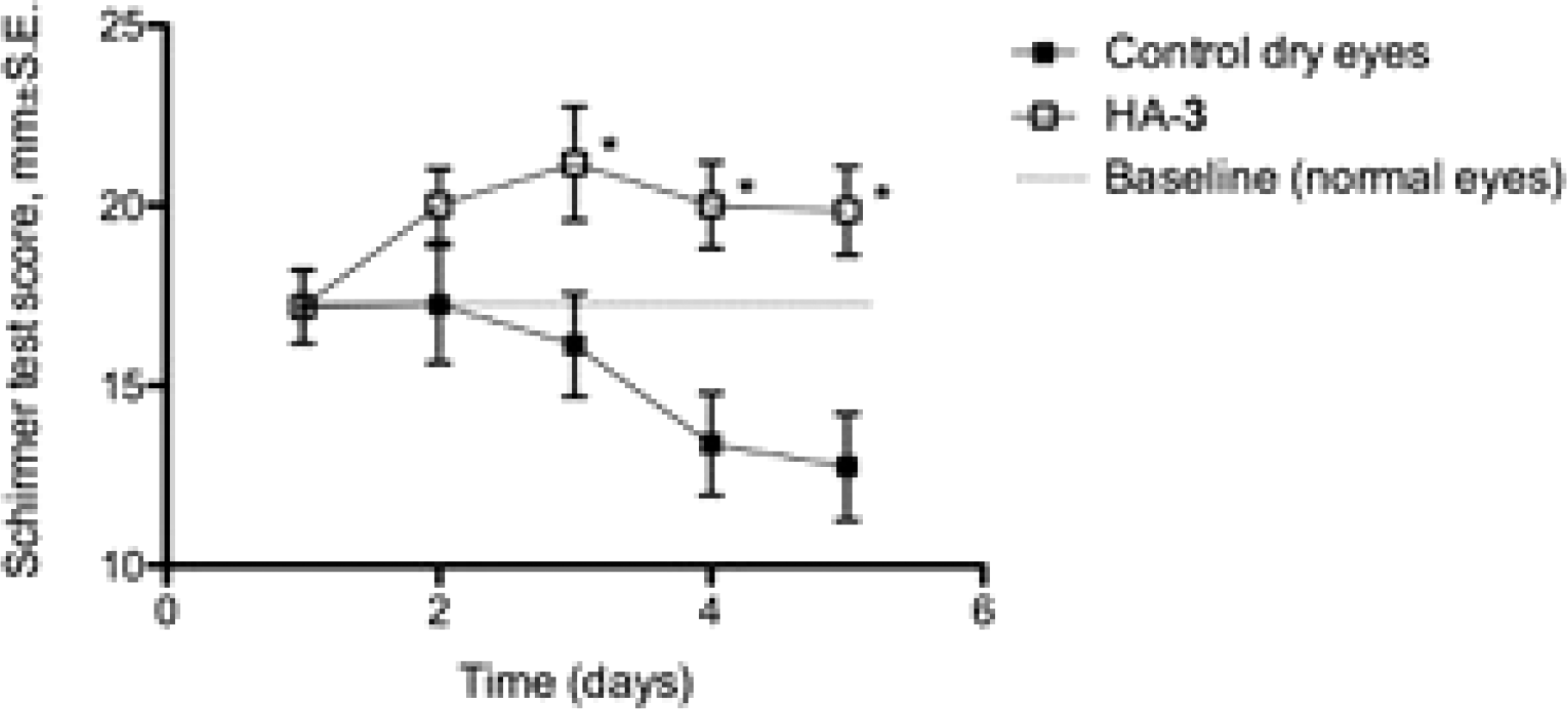
Schirmer test scores obtained using a 1 mg/mL solution of functionalized hyaluronic acid in the rabbit dry eye model (1 group, *n* = 8). *Significantly different from control dry eye (*p* < 0.05, unpaired *t* test with Welch’s correction).

The slit-lamp examination of the fluorescein-stained corneas revealed no occurrence of dotted staining in HA-**3** treated eyes (Figure S3, Supporting Information), differently from what happens in the control dry eyes where dry spots appear in 50.0 and 62.5% of the examined eyes on the fourth and fifth day, respectively. These results indicate that HA-**3** treatment protects against the appearance of defects of the corneal epithelium due to induced DES. In ophthalmology, HA is basically known as a product that keeps the ocular surface moistened,^31−33^ and its dispersions are considered the best artificial tear treatment. HA has a great capacity to bind water, protecting the corneal epithelium cells from desiccation. However, this effect is concentration dependent,^34^ and only polymeric concentrations higher than 0.15% significantly reduce the dry eye diseases. A role in ocular MMP expression has never clearly been demonstrated for HA: in many papers the protective effects of HA toward the corneal epithelium are reported, but these are limited to a good wetting.^33,35−38^ Recently, this effect has been well analyzed by some authors, on different experimental models and also on humans, who found that the HA-based artificial tears were able to keep the ocular surface well hydrated but not able to prevent the appearance of corneal areas of fluorescein uptake.^36,39,40^ Conversely, the functionalized hyaluronic acid under investigation is not only able to prevent corneal dryness but also corneal fluorescein staining. This behavior is attributable to its conjugation with the MMP inhibitor, whose activity in DES had already been proven in a previous study.^18^ After HA addition, the molecule still manifests to protect the cornea against the appearance of AS-induced dry spots (see Figure S3, SI).

The influence of the presence of HA on the wetting and mucoadhesive properties of the solution used on the ocular surface was investigated by means of contact angle measurements, as these can well detect both the properties. A thin layer of mucin in solid form was used as substrate so that both the wettability of the surface by the solution and the possible contribution of mucoadhesive interactions to the measured value could be evaluated. A contact angle value of 44.91° (SE ± 0.41) was measured for the MMP inhibitor solution, showing that it already has a good wetting capability due to the presence of a saline solution as the vehicle. The value decreased to 42.09° (SE ± 0.59) and 42.92° (SE ± 0.63) for the solutions containing HA and functionalized HA-**3**, respectively, with statistically significant differences (*p* < 0.05, unpaired *t* test; *n* = 10). This phenomenon can be attributed to the mucoadhesive interactions that hyaluronic acid is able to establish with the substrate of mucin. After all, hyaluronic acid is known as a polymer with good mucoadhesive properties,^41^ and the mucoadhesion is a phenomenon highly linked to the wetting abilities of the mucoadhesive toward the mucous substrate; indeed, according to the *wetting theory*, the wettability by the polymeric dispersion (and then its spreading ability) has a primary importance in establishing mucoadhesive interactions.^42,43^

Thanks to these characteristics and to the considerable hydrogen bonding ability of the corneal epithelial surface,^44^ it is reasonably possible that the presence of HA extends the residence time of the MMP inhibitor in the precorneal area, producing a longer-lasting activity. Further studies will aim to evaluate the correct dosage of the product, especially about the number of required daily administrations.

It can be concluded that the functionalized hyaluronic acid HA-**3** is able to maintain the activities of both its components after ocular administration: preservation of the integrity and hydration of the corneal surface in the induced DES model.

These unprecedented results open a new way for DES treatments and increase the interest in HA to counteract inflammation-induced tissue degradation.

## Supporting Information Available

Materials and methods for *in vitro* and *in vivo* tests, synthesis of **3** and of HA-**3**, Figure S1 and Figure S2 (PDF)
