# Supplementary material for: Functionalized Hyaluronic Acid for “In Situ” Matrix Metalloproteinase Inhibition: A Bioactive Material to Treat the Dry Eye Sydrome
Source: ACS Macro Lett. 2022 Sep 14;11(10):1190–4. doi: 10.1021/acsmacrolett.2c00455 (PMC9583614; doi:10.1021/acsmacrolett.2c00455)
Supplement: Supplementary file 1 — mz2c00455_si_001.pdf [file mz2c00455_si_001.pdf]

Supporting Information

Functionalized Hyaluronic Acid for "In situ" Matrix Metalloproteinases Inhibition: A Bioactive Material to Treat the Dry Eye Syndrome.

Susi Burgalassi,[¢] Marco Fragai,[¥,¦] Oscar Francesconi,[¥] Linda Cerofolini,[§] Daniela Monti,[¢] Gemma Leone,[§] Stefania Lamponi,[§] Giuseppe Greco,[¤] Agnese Magnani[§,]* and Cristina Nativi[¥,]*.

[¢]Department of Pharmacy, University of Pisa, via Bonanno 6, 56126 Pisa, Italy; [¥]Department of Chemistry (DICUS), University of Florence, Sesto Fiorentino, 50019 Italy; [¦]CeRM, via Sacconi, 6 Sesto Fiorentino, 50019 Italy; **;** *[§]CIRMMP, University of Florence, via Sacconi, 6 Sesto Fiorentino, 50019 Italy; [§]Department of Biotechnology, Chemistry and Pharmacy, via A. Moro, 2 53100 Siena, Italy; [¤]Rugani Hospital SR222 Chiantigiana, 53035 Colombaio (Siena) Italy.*

Table of Contents

Synthesis of inhibitor **3** Pag. S2
Synthesis of HA-**3** S2
Figure S1 S4
Human corneal epithelial cells – in vitro tests S4
Materials
Cytotoxicity test
Prevention of dehydration of human corneal epithelial cells
Evaluation of cell viability: NRU
Statistical analysis
Cytotoxicity test
Figure S2 S7
Wetting capability and in vivo tests S7
Materials & Formulations
Animals
Induction and treatment of dry eye conditions
Wetting capability: contact angles measurements
Figure S3 S9
Enzymatic inhibition assay S9
References S9

Synthesis of inhibitor ***3***

A solution of glycine methyl ester **4** in dichloromethane was cooled to 0° C and treated with triethyl amine. After 30 min, 4-(dimetilamino)pyridine (DMAP) and 4-methoxybenzensulfonyl chloride were added and the solution stirred at room temperature for 8 h. The crude sulfonamide so obtained (yellow solid, 97%) was dissolved in dry THF, hexanol **5** and Ph_3P were added to the solution, and the reaction mixture was slowly treated with diisopropyl azodicarboxylate (see Scheme S1). The reaction was completed in 18 h at room temperature and the crude **6** was purified by flash column chromatography on silica gel (87%). Treatment of **6** with hydroxylamine and KOH in MeOH/H_2O as solvent at room temperature (1h) and heated to 40 °C (6h) afforded the hydroxamic acid **7** as white solid (59%). Final removal of Boc protecting group with TFA (18h, rt) produced the desired derivatives **3** (87%).[S1]

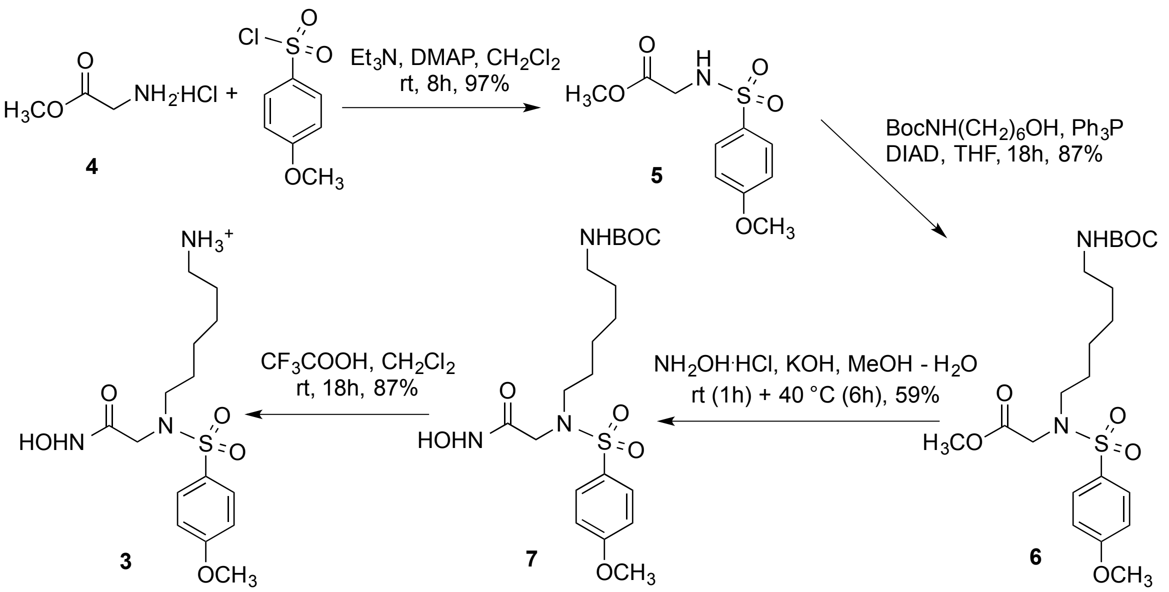

Scheme S1. Synthesis of MMP inhibitor **3**.

Synthesis of HA-3

Sodium hyaluronate was solubilized in bidistilled water and the aqueous solution was mixed under stirring. After 3 min of vigorous stirring, 1-ethyl-3-3 (dimethylaminopropyl) carbodiimide hydrochloride (EDC) and *N*-hydroxy-succinimide (NHS) and the MMP inhibitor 3 molecule were added (molar ratio: HA: MMP inhibitor 3: EDC: NHS 1:1:10:10). The reaction was left at 25° C, under stirring for 2 h; subsequently, absolute ethanol (400 mL) was added and the solution left at 4°

C overnight. The precipitate was dissolved in bidistilled water and the solution was dialyzed for 48 h and freeze-dried.

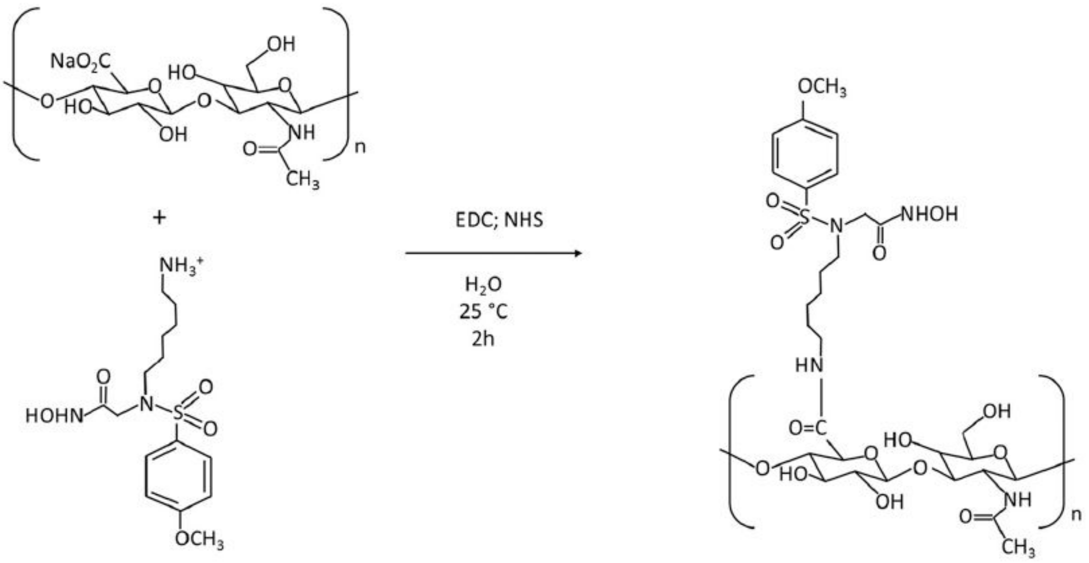

Scheme S2. Synthesis of HA functionalized with MMP inhibitor 3, HA-3.

Enzymatic tests pointed out that after 3 h, HA-**3** showed a 10% increased stability to in vitro enzymatic degradation in comparison to native HA, resistance that increased up to about 50% after 24 h (see also ref. 28 in the main text).[S1]

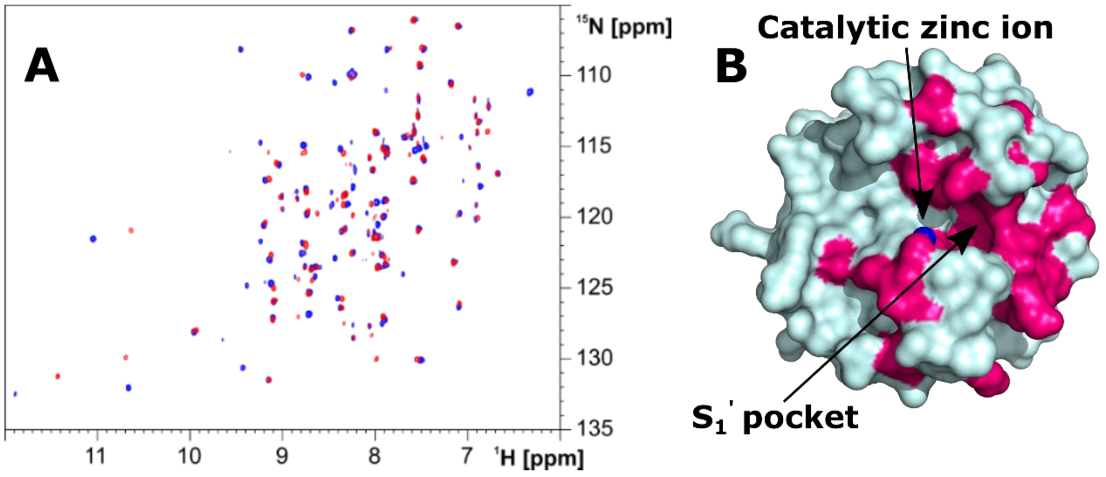

Figure S1. Superimposed ^{1}H-^{15}N HSQC spectra of MMP-12 catalytic domain (blue cross-peaks) and of its complex with the inhibitor **3** (red cross-peaks). The spectra were recorded on a sample of MMP-12 at the concentration of 0.1 mM. The complex was obtained by adding an equimolar concentration of inhibitor **3** to the sample of the protein. Surface representation of the catalytic domain of MMP-12 showing the S1' pocket and the catalytic zinc ion. The residues experiencing the largest chemical shift perturbation in the presence of the inhibitor **3** are colored in magenta.

Human corneal epithelial cells – in vitro tests

Materials

EpiGRO™ Human Corneal Epithelial Cells, EpiGRO ocular epithelia complete media kit. Dulbecco's Modified Eagle's Medium (DMEM), trypsin solution, and all the solvents used for cell culture were purchased from Sigma-Aldrich (Milan, Italy). Mouse immortalized fibroblasts NIH3T3 were from American Type Culture Collection (USA). Positive control, PVC-org. Sn, was supplied from US Pharmacopeia, Rockville, MD USA.

Cytotoxicity test

In order to evaluate the in vitro cytotoxicity of new products, the direct contact tests was used [S2]. This test is suitable for sample with various shapes, sizes or physical status (i.e., liquid or solid). The evaluation of in vitro cytotoxicity does not depend on the final use for which the product is intended, and the document ISO 10995-5:2009 recommends many cell lines from American Type Collection. Among them, NIH3T3 mouse fibroblasts were chosen [S3].

NIH3T3 fibroblasts were propagated in DMEM supplemented with 10% fetal calf serum, 1% L-glutamine-penicillin-streptomycin solution, and 1% MEM non-essential amino acid solution, and incubated at 37°C in a humidified atmosphere containing 5% CO2. Once at confluence, the cells were

washed with PBS 0.1M, separated with trypsin-EDTA solution and centrifuged at 1000 r.p.m. for 5 minutes. The pellet was re-suspended in complete medium (dilution 1:15). Cells (8.0 x 104) suspended in 1 ml of complete medium were seeded in each well of a 24 well round multidish and incubated at 37°C in an atmosphere of 5% CO2. Once reached the 50% of confluence (i.e., after 24 hours of culture), the culture medium was discharged and the test compounds, sodium hyaluronate (HA) and HA-MMi, properly diluted in completed medium (1.3x10-7 M) and filtered by 0.22 µm pores syringe filters, were added to each well. The cytotoxicity test was repeated three times and all samples were set up in five replicates. Complete medium was used as negative control and PVC-org. Sn as positive control. After 24 hours of incubation, cell viability was evaluated by Neutral Red Uptake (NRU) assay [S4].

Prevention of dehydration of human corneal epithelial cells

Human corneal epithelial cells (HCECs) were propagated in appropriated EpiGRO medium and incubated at 37°C in a humidified atmosphere containing 5% CO_2. Once at confluence, the cells were washed with PBS 0.1M, separated with trypsin-EDTA solution and centrifuged at 1000 r.p.m. for 5 minutes. The pellet was re-suspended in complete basal EpiGRO medium (dilution 1:15). Cells (8.0 x 104) suspended in 1 ml of basal medium were seeded in each well of a 24 well round multidish and incubated at 37°C in an atmosphere of 5% CO_2. Once reached the 80% of confluence, the cells were washed 3 times with 100 µL of PBS (1x), and the test compounds, properly diluted in PBS buffered in order to have at pH = 7.2 was added to each well. The concentration tested of HA-**3** was 0.15% in HA, i.e., the same concentration value of HA in OPTO yal A tear substitute (SOOFT Italia S.p.A., Fermo, Italy). PBS buffered at pH = 7.2 was used as negative control and OPTO yal A as positive control. The cells in contact with the test samples were then incubated for 20 minutes at 37 °C in an atmosphere containing 5% CO_2. At the end of the incubation, the solutions under examination were removed, the cells were washed with 100 µL of PBS (1x) and subjected to a continuous flow of air for 0 and 30 minutes at room temperature. Finally, cell viability was evaluated by Neutral Red Uptake (NRU) assay [S4]. Each sample was tested in triplicate.

Evaluation of cell viability: NRU

To determine the percentage of viable cells as follows, the following solutions were prepared:

1. Neutral Red (NR) stock solution: 0.33 g NR dye powder in 100 ml sterile H_2O

2. NR medium: 1.0 ml NR stock solution+99.0 routine culture medium pre-warmed to 37°C

3. NR desorb solution: 1% glacial acetic acid solution + 50% ethanol + 49% H_2O

At the end of incubation, the routine culture medium was removed from each plate and the cells were carefully rinsed with 1 ml pre-warmed D-PBS 0.1M. Plates were then gently blotted with paper towels. 1.0 ml NR medium was added to each dish and further incubated at 37°C, 95% humidity, 5.0% CO_2 for 3 hours. The cells were checked during incubation for NR crystal formation. After incubation, the NR medium was removed, and the cells were carefully rinsed with 1 ml pre-warmed D-PBS 0.1M. PBS was decanted and blotted from the dishes and exactly 1 ml NR desorb solution was added to each sample. Plates were placed on a shaker for 20-45 minutes to extract NR from the cells and form a homogeneous solution. During this step the samples were covered to protect them from light. Five minutes after removal from the shaker, absorbance was read at 540 nm with a UV/visible spectrophotometer (Pharmacia Biotech Ultrospec 2000).

Statistical analysis

Multiple comparisons were performed by one-way ANOVA and individual differences tested by Fisher's test after the demonstration of significant intergroup differences by ANOVA. Differences with $p < 0.05$ were considered significant.

Cytotoxicity test

Non-confluent adhered mouse fibroblasts NIH3T3 were incubated with HA and HA-**3** at a concentration of 1.3x10-7 M. Cells were analyzed after 24 hours of contact with the test samples and the results are reported in Fig.1. As shown, the percentage of viable fibroblasts in contact with HA-**3** is significantly higher in comparison to both HA and negative control (complete medium). So, not only that HA-**3** has no cytotoxic effect but is also able to enhance the proliferation of NIH3T3

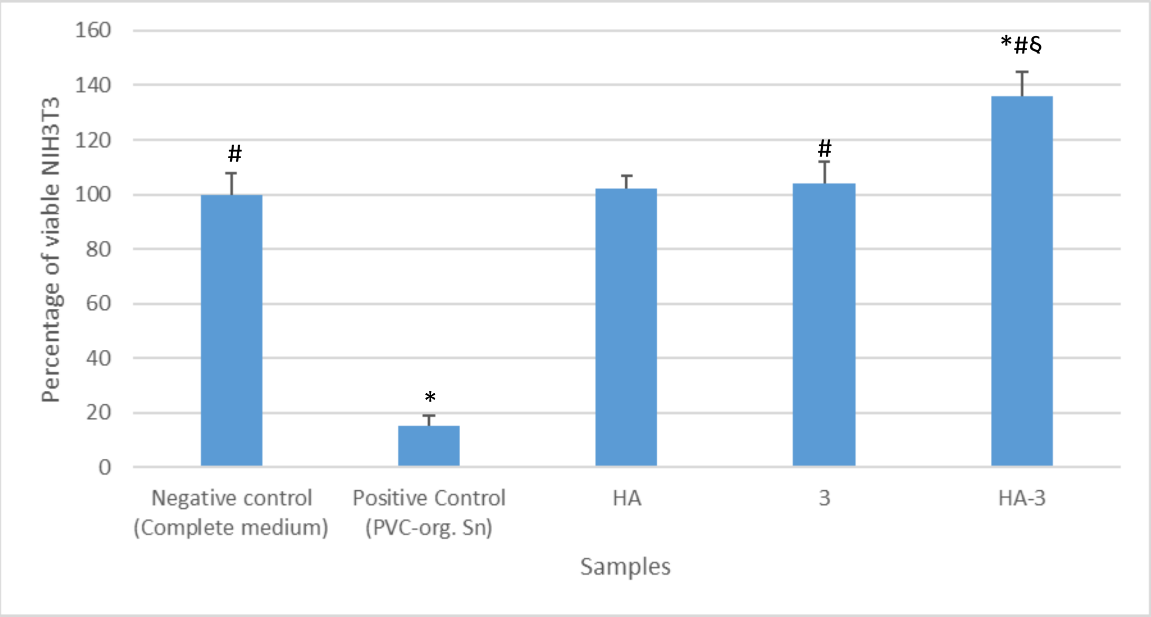

Figure S2. Percentage of viable NIH3T3 after 24 hours of contact with HA, HA-MMPi (HA-**3**), negative control (complete medium) and positive control (PVC-org. Sn) as determined by the Neutral Red Uptake. Data are mean ± SD three experiments performed in five replicates. *Values are statistically different versus negative control (complete medium), $p < 0.05$. #Values are statistically different versus positive control (PVC-org. Sn), $p < 0.05$. § Value is statistically different versus percentage of viable cells in contact with HA, $p < 0.05$.

Wetting capability and in vivo tests

Materials and formulations

Hog gastric mucin (HGM; Carl Roth GmbH+Co. KG, Karlsruhe, Germany).
Solutions containing 1 mg/mL of HA-MMP inhibitor (HA-**3**), MMP inhibitor (**3**) or HA in physiological solution (NaCl, 0.9%) were used for the tests.
For in vivo tests, the HA-**3** solution was filtrated by a 0.45 μm membrane (Minisart, Sartorius SpA, Florence, Italy) and used immediately.

Animals

Non-anaesthetized, male, New Zealand albino rabbits, weighing 2.5-3.0 Kg (Pampaloni Rabbitry, Fauglia, Italy) were used and treated as prescribed in the publication "Guide for the Care and Use of Laboratory Animals".[S5] All experiments conformed with the ARVO Resolution on the Use of Animals in Research: they were carried out under veterinary supervision, and after approval of the protocols by the Ethical-Scientific Committee of the University of Pisa.
The animals were housed singly in standard cages in a room with controlled lighting, at 19 ± 1°C and 50 ± 5% R.H., with no restriction of food or water. During the experiments, the rabbits were placed in restraining boxes to which they had been habituated, in a room with dim lighting; they were allowed to move their heads freely, and their eye movements were not restricted.

Induction and treatment of dry eye conditions

The animals were preliminarily submitted to the Schirmer I test [S6] and to slit-lamp examination of the corneal surface to verify the integrity of the corneal epithelium and the function of lachrymal system; then eight of them were treated as reported in Burgalassi et al. [S7] Briefly, the animals received in the lower conjunctival sac of both eyes 50 μl of 1%

atropine solution (AS) at 8.00 a.m., 1.00 p.m. and 6.00 p.m. Five minutes after each administration of AS they received in one eye 50 μl of the formulation under study, while vehicle alone was administered in the contralateral (control) eye. All treatments were discontinued after 5 days.

The Schirmer I test was performed 2, 3, 4 and 5 days after the first administration of AS, at 10.00 a.m. The test was performed on both eyes (non-anaesthetized) of all animals, by maintaining for 3 min a standardized test strip (Alfa Intes, Casoria, Italy) into the external third of the lower conjunctival fornix. The wetted length in millimeters of the strip was taken as the test score.

After staining with fluorescein (Fluorets, Smith & Nephew Pharmaceuticals Ltd, Romford, UK), the corneal surface was observed by slit-lamp biomicroscope fitted with a blue filter. The test was performed at 2.00 p.m., 3, 4 and 5 days after the first administration of AS. The occurrence of dotted staining, revealing the presence of dry spots on the ocular surface, was considered as a symptom of corneal desiccation.

Wetting capability: Contact angle measurements

The wettability characteristics of a mucous substrate by the test solutions were analyzed by contact angle measurements. An optical contact angle-measuring instrument (OCA 15, DataPhysics Instrument GmbH, Germany) was used to determine static contact angles of the solutions on a thin tablet of HGM (20 mg) produced by hydraulic press fitted with 60 mm flat punches applying a compression force of 19600 N (Perkin-Elmer) for 30 seconds. The system consisted of a high-resolution CCD video camera and a six-fold power zoom lens with integrated fine focusing; the images of the drops were recorded and analyzed by SCA 20 software. The sessile drop method was used: briefly, it consists of placing a known volume of solution test on the HGM tablet. When the spreading of the droplet attains an equilibrium state, the contact angle is determined. Ten measurements were made for each test solution.

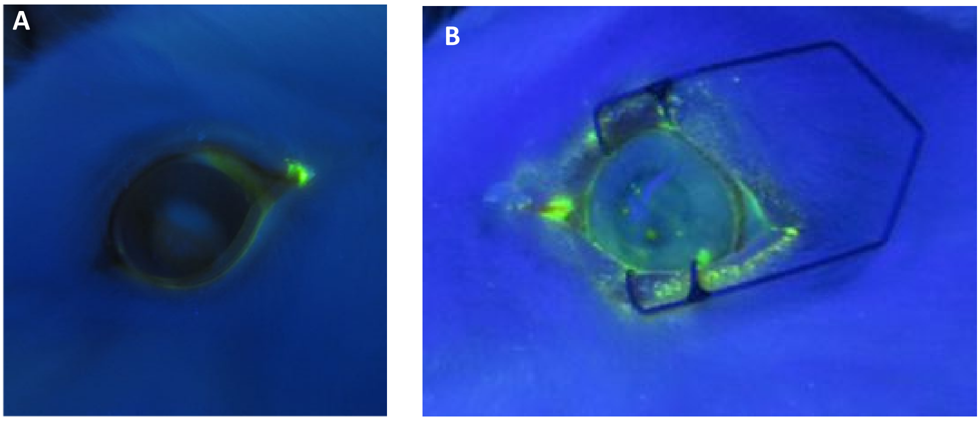

Figure S3. Images of rabbit eye after staining with fluorescein. A: HA-**3** treated, no stained spots are observed on the corneal surface; B: control eye, some dry spots are evident on the corneal surface.

Enzymatic inhibition assay

All measurements were performed at 298 K in a 50 mM HEPES-buffered solution, with 10 mM $CaCl_2$, 0.05% Brij- 35 and 0.1 mM $ZnCl_2$ (pH 7.0), by using 1 nM of MMP-9 and 1 μM of fluorescent peptide (OmniMMP fluorogenic substrate; Enzo Life Science, Farmingdale, NY, USA). The fluorescence (excitationmax 328 nm; emissionmax 393 nm) was measured for 3 minutes after the addition of the substrate by using a Varian Eclipse fluorimeter. Fitting of rates as a function of compound **3** concentration provided the K_i values.

References

[S1] Leone, G.; Pepi, S.; Consumi, M.; Lamponi, S.; Fragai, M.; Martinucci, M.; Baldoneschi, V.; Francesconi, O.; Nativi, C.; Magnani, A. Sodium Hyaluronate-g-2-((N-(6-Aminohexyl)-4-Methoxyphenyl)Sulfonamido)-N-Hydroxyacetamide with Enhanced Affinity towards MMP12 Catalytic Domain to Be Used as Visco-Supplement with Increased Degradation Resistance. Carbohydrate Polymers 2021, 271. https://doi.org/10.1016/j.carbpol.2021.118452.

[S2] ISO 10995-5:2009; Biological evaluation of medical devices – Part 5: Tests for cytotoxicity: in vitro methods. https://www.iso.org/standard/36406.html

[S3] Lamponi, S.; Leone, G. Consumi, M.; Greco, G.; Magnani, A. In vitro biocompatibility of new PVA-based hydrogels as vitreous body substitutes. J Biomater Sci Polym Ed. 2012,23, 555-575.DOI:10.1163/092050611X554499

[S4] Lamponi, S.; Leone, G.; Consumi, M.; Greco, G.; Nelli, N.; Magnani, A. Porous multi-layered composite hydrogel as cell substrate for in vitro culture of chondrocytes. Int J Polym Mater 2020, DOI: 10.1080/00914037.2020.1765351

[S5] NIH Publication No. 92-93, revised 1985

[S6] Schirmer O.; Studien zur physiologie und pathologie der tränenabsonderung und tränenabfuhr. Arch Klin Ophthalmol 1903; 56:197-291

[S7] Burgalassi S, Panichi L, Chetoni P, Saettone MF, Boldrini E. Development of a simple dry eye model in the albino rabbit and evaluation of some tear substitutes. Ophthalmic Res 1999; 31:229-235.
